# PARP1 mediated PARylation contributes to myogenic progression and glucocorticoid transcriptional response

**DOI:** 10.1038/s41420-023-01420-2

**Published:** 2023-04-22

**Authors:** Arnold Tan, Awais Z. Younis, Alexander Evans, Jade V. Creighton, Clare Coveny, David J. Boocock, Craig Sale, Gareth G. Lavery, Amanda S. Coutts, Craig L. Doig

**Affiliations:** 1 School of Science and Technology, Department of Biosciences, Nottingham Trent University, Nottingham, NG11 8NS UK; 2 John van Geest Cancer Research Centre, Nottingham Trent University, Nottingham, NG11 8NS UK; 3 Institute of Sport, Manchester Metropolitan University, Manchester, M1 7EL UK

**Keywords:** DNA repair enzymes, Mechanisms of disease

## Abstract

The ADP-ribosyltransferase, PARP1 enzymatically generates and applies the post-translational modification, ADP-Ribose (ADPR). PARP1 roles in genome maintenance are well described, but recent work highlights roles in many fundamental processes including cellular identity and energy homeostasis. Herein, we show in both mouse and human skeletal muscle cells that PARP1-mediated PARylation is a regulator of the myogenic program and the muscle transcriptional response to steroid hormones. Chemical PARP1 modulation impacts the expression of major myocellular proteins, including troponins, key in dictating muscle contractile force. Whilst PARP1 in absence of DNA damage is often assumed to be basally inactive, we show PARylation to be acutely sensitive to extracellular glucose concentrations and the steroid hormone class, glucocorticoids which exert considerable authority over muscle tissue mass. Specifically, we find during myogenesis, a transient and significant rise in PAR. This early-stage differentiation event, if blocked with PARP1 inhibition, reduced the abundance of important muscle proteins in the fully differentiated myotubes. This suggests that PAR targets during early-stage differentiation are central to the proper development of the muscle contractile unit. We also show that reduced PARP1 in myoblasts impacts a variety of metabolic pathways in line with the recorded actions of glucocorticoids. Currently, as both regulators of myogenesis and muscle mass loss, glucocorticoids represent a clinical conundrum. Our work goes on to identify that PARP1 influences transcriptional activation by glucocorticoids of a subset of genes critical to human skeletal muscle pathology. These genes may therefore signify a regulatory battery of targets through which selective glucocorticoid modulation could be achieved. Collectively, our data provide clear links between PARP1-mediated PARylation and skeletal muscle homeostatic mechanisms crucial to tissue mass maintenance and endocrine response.

## Introduction

PARP1 irreversibly cleaves NAD^+^, producing nicotinamide for re-salvage and monomeric ADP-Ribose (ADPR) units. ADPR functions as a signaling moiety through site-specific attachment to target molecules, altering their biological activity. ADPR units can be mono- or poly-elongated: the latter produces poly-ADPR (PAR) chains, in a process termed PARylation [1]. To date, PARP1, alongside PARP2 and the tankyrases, are capable of performing PARylation. Recent studies of PARylation have revealed roles ancillary to the well-characterized genome repair. For example, PARP1 activity has been shown to govern fundamental processes including adipogenesis, RNA stability, and transdifferentiation [2–6].

The conserved and constitutive nature of PARP1 and PARylation underlies the variety of documented actions. Emerging studies implicate PARP1 within skeletal muscle metabolism and myogenesis [4, 7–9]. For example, in myogenic progression, downregulation of PARP1 in fully formed myotubes is required for oxidative stress resistance [9]. More recent work has demonstrated *PARP1* binding to regulatory regions of the *MYOD* target muscle genes *p57* and *myogenin* [4]. These works demonstrate PARP1 also exerts myogenic influence independent of its PARylating activity, however, the impacts of PARylation in skeletal muscle remain poorly characterized. Despite this incomplete understanding, PARP inhibitors have been suggested for alleviation of inflammation in non-communicable chronic diseases including myopathy.

Herein, we used transcriptomic and proteomic analyses to explore PARP1-mediated PARylation during myogenic progression. We show PARP1 and the PAR it applies during myogenesis are dynamic, and sensitive to both hormonal status and metabolic demand. Early-stage muscle cell differentiation sees a transient rise in PARylation that holds influence over the fully developed myotube. This indicates that the molecular targets of PARP1 during early-stage differentiation are pivotal in determining the functional quality of the muscle fiber. We also demonstrate that PARP1 holds impacts over the skeletal muscle transcriptional response to glucocorticoids, steroid hormones with governance over muscle protein synthesis and metabolic rate. We identify a subset of genes that are critical to muscle mass and contractile function that are co-regulated by PARP1. These results reveal whilst PARP1 inhibition mediates beneficial effects in fully developed muscle tissue, the potential for negative impacts exists for muscle differentiation and steroid hormone activation. Therefore, the use of clinically available PARP inhibition should be subject to greater consideration for impacts on the whole-body muscle mass.

## Results

### PARP1-mediated PARylation is dynamic during skeletal muscle differentiation

**Fig. 1:**
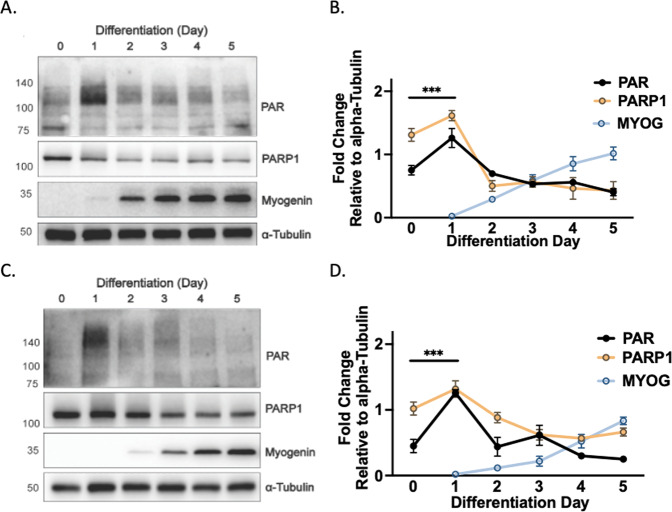
PARP1 and PARylation are dynamic during myogenesis. **A** Western immunoblotting of differentiating C2C12 myoblasts probed for PARylation (PAR), PARP1, Myogenin, and Alpha-Tubulin (representative of *n* = 4). **B** Quantification of MYOG, PAR, and PARP1 present over differentiation. Each bar represents means ± S.D (*n* = 4) ***P < 0.001. **C** Western immunoblotting of differentiating LHCN-M2 human myoblasts probed for MYOG, PAR, PARP1, Myogenin, and Alpha-Tubulin (representative of *n* = 4). **D** Quantification of PAR and PARP1 present over differentiation. Each bar represents means ± S.D (*n* = 4) ***P < 0.001.

Molecular assessment of PARP1 and PAR during myogenesis remains limited. Given PARP1 non-enzymatically regulates the myogenic regulatory factor *MYOD* [4], we hypothesized that enzymatically driven PARylation also contributes to myogenic transition. To address this, C2C12 myoblasts were differentiated and interrogated for PAR and PARP1 levels on each day of differentiation. We report that levels of PAR were dynamic during myoblast alignment and fusion, reaching peak within day 1 and nadir by day 5 of differentiation (Fig. 1A, B). Identical dynamic regulation of PAR and PARP1 abundance was recorded in the human muscle cell line LHCN-M2 (Fig. 1C, D). Total levels of myogenin also increased, demonstrating the establishment of myogenic commitment in these myoblasts (Fig. 1A, C). Together, these data indicate, at least in mammals, a conserved process of PAR regulation driven by PARP1 that is associated with the initiation of the myogenic program.

### PARylation in skeletal muscle myogenesis is metabolically sensitive

**Fig. 2:**
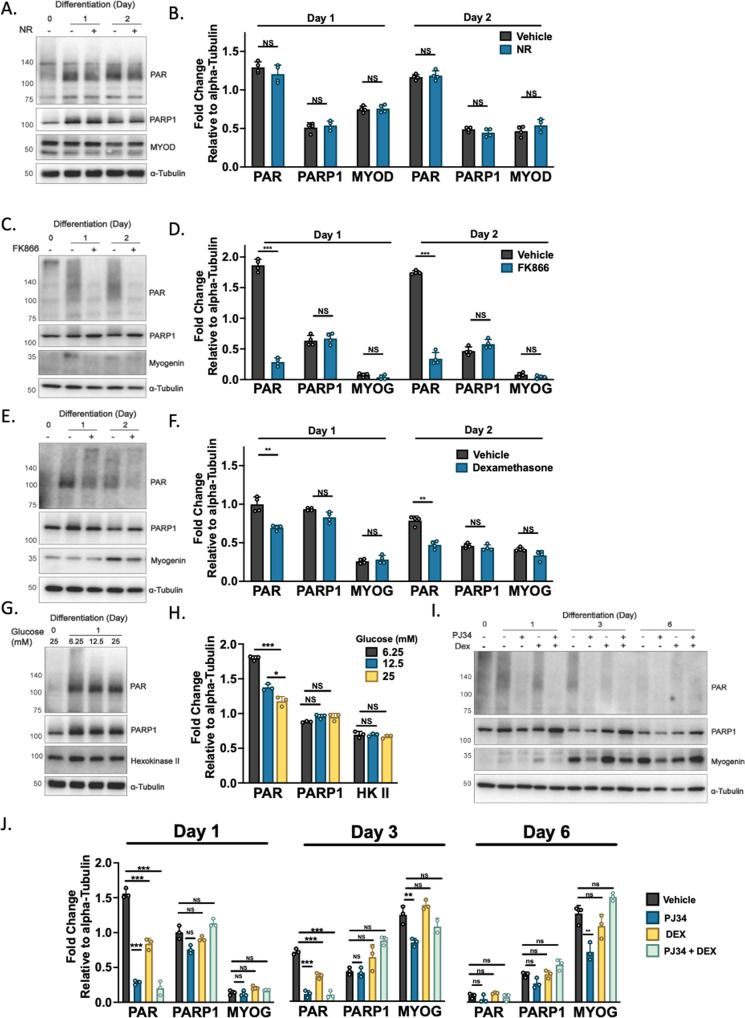
PARP1 and PARylation in differentiating myoblasts are sensitive to changes in metabolism. **A**, **B** Western immunoblotting of differentiating myoblasts differentiated in ±nicotinamide riboside (NR) (0.5 mM) (representative of *n* = 4) probed for PAR, PARP1, MYOD, and Alpha-Tubulin. **C**, **D** Western immunoblotting of differentiating myoblasts differentiated in ± NAMPT specific inhibitor FK866 (50 nM) (representative of *n* = 4) probed for PAR, PARP1, Myogenin, and Alpha-Tubulin, ***P < 0.001. **E**, **F** Western immunoblotting of differentiating myoblasts differentiated in ±dexamethasone (1 µM) probed for PAR, PARP1, MYOD and Alpha-Tubulin (representative of *n* = 4, **P < 0.01). **G**, **H** C2C12 myoblasts differentiated in differentiation medium containing different glucose concentrations before lysate harvest and immunoblotted for PAR, PARP1, Hexokinase II, and Alpha-Tubulin (representative of *n* = 3, *P < 0.05). **I**, **J** Western immunoblotting of differentiating myoblasts differentiated in ±dexamethasone (1 µM), PARP inhibitor PJ34 (10 µM) or both in combination (representative of *n* = 3, *P < 0.05, **P < 0.01).

Conventionally, PARP1 is regarded as inactive in the absence of genotoxic stress. However, several studies have demonstrated that both PARP1 and PARylation are fundamental to a variety of biological processes [1]. As such, we explored the response of PARP1 and PAR to conditions of NAD^+^ excess or deficit during myogenesis. The NAD^+^ precursor nicotinamide riboside (NR) is bioavailable to human skeletal muscle and can directly generate NAD^+^ [10]. However, we find that NR supplementation of myoblasts during differentiation induction did not alter the levels of day 1 PAR accumulation (Fig. 2A, B). In contrast, we find NAD^+^ depletion through specific NAMPT inhibition with FK866 reduced day 1 PAR levels and myogenin expression, supporting an association of the enzymatic product of PARP1 with myogenic progression (Fig. 2C, D). We also found PAR during myogenesis to be regulated by the external cellular environment, with a dose-dependent increase in PAR in response to glucose deprivation (Fig. 2G, H) and is sensitive to steroid hormone levels following exposure to the glucocorticoid receptor (GR) agonist dexamethasone (Fig. 2E, F). Collectively, these data show that PARP1-mediated PARylation is active in differentiating myoblasts and exerts plasticity. Moreover, we find a synergistic interaction of both the inhibition of PARP1 with the inhibitor PJ34 and dexamethasone treatment during differentiation induction upon subsequent PAR abundance (Fig. 2I, J). Together, these data show PARP1 and PAR in skeletal muscle to be basally detected and responsive to the changing cellular environment. This implies PAR in skeletal muscle is a signaling motif with potential roles to play in the variety of pathways governing muscle homeostasis.

### Modulation of PARylation during early-stage myogenesis

**Fig. 3:**
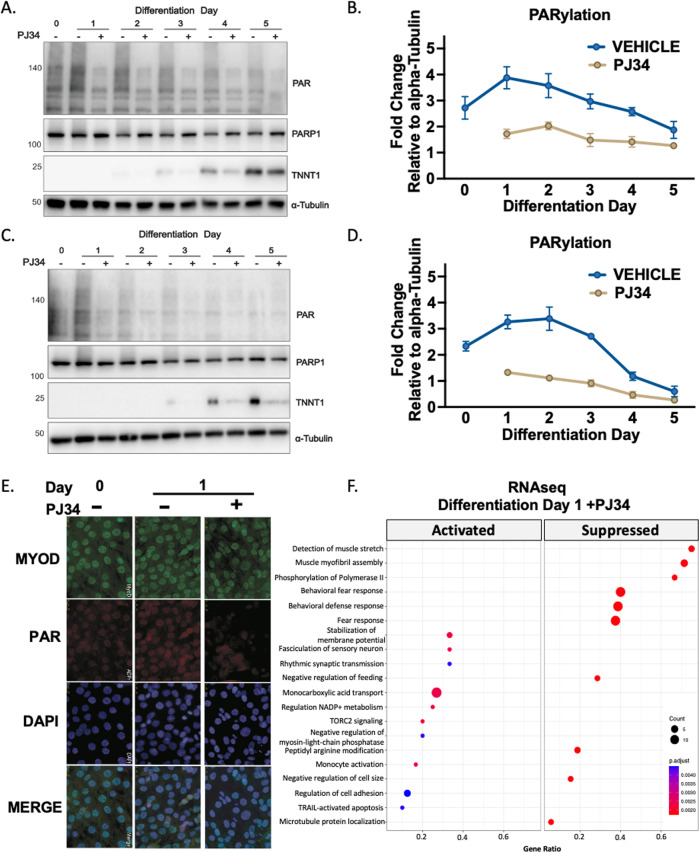
Modulation of PARylation during myogenesis. **A** Western immunoblotting of differentiating C2C12 myoblasts differentiated in ±PARP inhibitor PJ34 (10 µM) probed for PAR, PARP1, Troponin 1 (TNNT1), and Alpha-Tubulin (representative of *n* = 4). **B** Quantification of PAR and PARP1 during differentiation. Each bar represents means ± S.D (representative of *n* = 4). **C** Western immunoblotting of differentiating LHCN-M2 myoblasts differentiated in ±PARP inhibitor PJ34 (10 µM) probed for PAR, PARP1, TNNT1, and Alpha-Tubulin (*n* = 4) *P < 0.05, **P < 0.01, and ***P < 0.001. **D** Quantification of PAR and PARP1 in human myoblasts during differentiation. Each bar represents means ± S.D (representative of n = 4). **E** Immunostaining of PAR (red), DAPI (purple), and MYOD (green) in differentiating C2C12 myoblasts (*n* = 4). **F** Dot plot of gene ontology (GO) overrepresentation analysis of C2C12 myoblasts differentiated in ±PARP inhibitor PJ34 (10 µM) (*n* = 3) on day 1 of differentiation. The *x* axis shows the gene ratio which represents the percentage of genes enriched in a term. The *y* axis represents the enriched pathways: size of the node represents the number of enriched genes in the term.

To modulate PAR generation, we applied the broad-spectrum PARP inhibitor PJ34 during differentiation induction and demonstrate significant reduction of early-stage PAR accumulation in both murine and human skeletal muscle cells (Fig. 3A–D). PJ34 treatment also significantly increased cellular NAD^+^ (Supplementary Fig. 1A). To further investigate if the transient day 1 PAR accumulation is a determinant of developed myotube functional quality, we also assessed levels of Troponin T1 (TNNT1), a subunit of the sarcomere. This was reduced in myotubes exposed to PJ34 during early-stage differentiation and suggests PARylation is required for proper thin filament assembly (Fig. 3A, C). These PAR trends were also replicated using Rucaparib (Supplementary Fig. 2A, B). Inhibition of PARP1 with PJ34 is also shown by immunofluorescence, with the day 1 PAR accumulation impaired in the PJ34-treated differentiating myoblasts (Fig. 3E). To identify the major transcriptional processes governed by PARP1 inhibition, we conducted RNAseq on PJ34 treated early-stage differentiating C2C12 myoblasts compared to vehicle controls (Fig. 3F). Gene Ontology analysis revealed suppression of pathways regulating assembly of the skeletal muscle myofibrils and detection of muscle stretch, a table of differentially expressed genes is provided (Supplementary Table S1). Additionally, in line with documented actions of PARP1 in transcriptional regulation [11] suppression of the phosphorylation of RNA polymerase II suggests global shifts in transcriptional rate as a result of reduced PARP1 activity. Collectively, these data confirm the dynamic nature of PAR in skeletal muscle and a background of transcriptional coordination during early-stage differentiation.

### Differentiating skeletal muscle and impacts of PARP1 upon the proteome

**Fig. 4:**
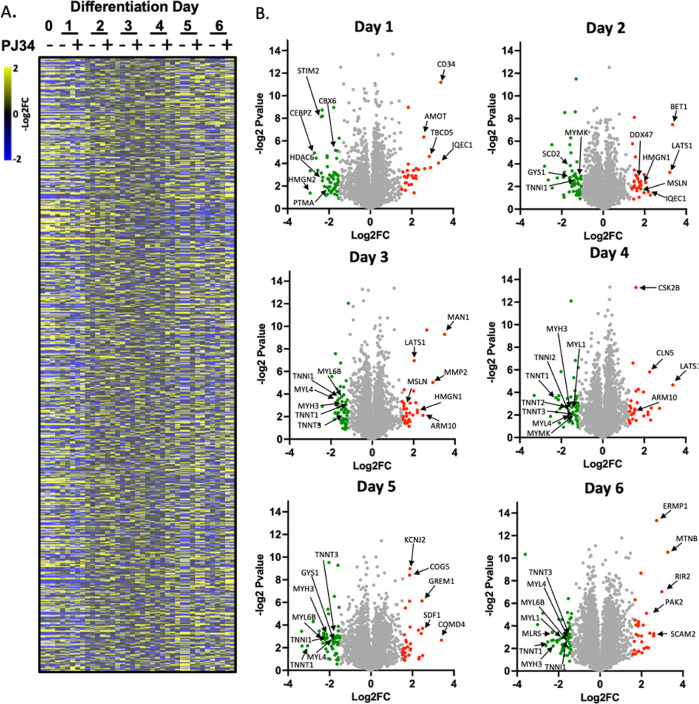
The PARP1-regulated proteome in differentiating skeletal muscle. **A** Heatmap showing 2911 detected proteins in differentiating C2C12 myoblasts differentiated in ± PARP inhibitor PJ34 (10 µM) over days 0, 1, 2, 3, 4, 5, and 6 of differentiation (*n* = 3 per condition). **B** Volcano plots showing differentially abundant proteins in presence of PJ34 on each day of the 6 days of differentiation. Downregulated proteins are green (>−1.5 fold change), upregulated are red (>1.5 fold change). Arrows denote named proteins.

To gain insight into proteomic changes during differentiation regulated by PARP1-generated PAR, we subjected lysates of differentiating myoblasts treated with PJ34 (Fig. 3A) to unbiased SWATH-MS analysis. These recovered peptides aligned with 2911 identified proteins expressed from day 0 to day 6 of differentiation ±PJ34 (Fig. 4A). Examination of PJ34 treated day 1 differentiating lysates revealed differentially abundant proteins associated with documented roles of PARP1 in chromosome biology, and downregulated proteins (>1.5 fold change) included CEBPZ (−2.70 fold change, Pvalue 0.04), CBX6 (−1.59 fold change Pvalue 0.05), STIM2 (−2.37 fold change, Pvalue 0.004) and CDN1B (−2.09 fold change Pvalue 0.03) (Fig. 4B). These data describe muscle PARP1 as dictating chromatin features, consistent with regulation of transcriptional programs across tissues [12]. Subsequently, downregulated proteins from PJ34 treated day 3 differentiating lysates shifted towards association with skeletal muscle functions including MYL4 (−1.68 fold change Pvalue 0.10), and TNNI1 (−1.60 fold change Pvalue 0.11) (Fig. 4B). Proteins fundamental to skeletal muscle phenotype and contractility also shift in PJ34 treated day 5 differentiating lysates, including TNNT1 (−3.35 fold change, Pvalue 0.05), INSR (-4.36 fold change *P* value 0.04). A tabular list of differentially abundant proteins is provided (Supplementary Table S2).

### Proper myoblast differentiation requires PARP1 activity

**Fig. 5:**
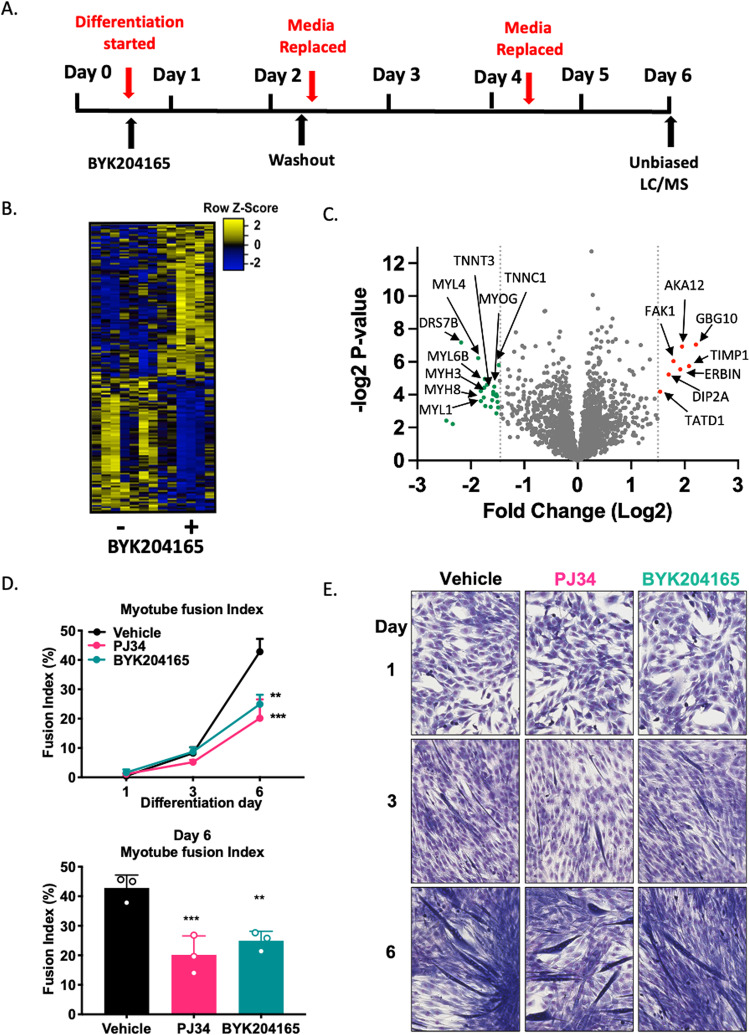
PARP1-mediated PAR events on Day 1 impact the myogenic trajectory. **A** Schematic representation of the PARP inhibitor treatment protocol during C2C12 myoblast differentiation. **B** Heatmap representing differential abundance of proteins within samples differentiated in ±PARP1 specific inhibitor BYK204165 (10 µM) (Vehicle *n* = 7, BYK204165 *n* = 6). **C** Volcano plot of SWATH-LCMS lysates recovered from day 6 differentiated C2C12 myoblasts differentiated in ± PARP inhibitor PJ34 (10 µM). Differential protein abundance shown with downregulated proteins marked green and upregulated proteins marked red. **D** Myotube fusion index of differentiating myoblasts differentiated in ±PARP inhibitor PJ34 (10 µM) or ±PARP1 specific inhibitor BYK204165 (10 µM) (*n* = 3). Cells were fixed on days 1, 3, and 6 of differentiation. Upper panel shows days 1, 3, and 6 fusion index, lower panel shows day 6 fusion index. Each bar represents means ± S.D (*n* = 3) ***P < 0.001, **P < 0.01. **E** Representative photographs of Jenner-Giemsa stained differentiating myoblasts over days 1, 3, and 6 of differentiation (*n* = 3).

PJ34 is a broad PARP inhibitor that can target other members of the PARP family of enzymes, including the ADP-ribosylating tankyrases [13]. Given this, we sought to explore the observed proteomic changes and challenge the extent of day 1 PARP1 mediated PARylation over myogenesis. To do this, we also treated C2C12 myoblasts with the highly specific PARP1 inhibitor BYK204165 [14] during differentiation induction. We assessed levels of PAR as well as subsequent impacts on differentiation trajectory and observed ablation in total PAR protein levels in BYK204165 treated groups (Supplementary Fig. S1B). Myoblasts were treated on day 1 with BYK204165 for 24 h before washout and subsequently left to differentiate for 6 days with media changed every 48 h (Fig. 5A). Lysates were harvested and subjected to unbiased SWATH-MS analysis. This detected 2921 proteins, of which 180 were significantly differentially expressed in BYK204165-treated differentiating lysates (Fig. 5B and Supplementary Table S3). Of the downregulated proteins, these again included muscle contractile proteins including TNNT1 (−1.31 fold change), MYL4 (−1.86 fold change), TNNT3 (−1.53 fold change), MYL1 (−1.81 fold change) and MYH3 (−1.83 fold change) (Fig. 5C). Pathway overrepresentation analysis of these samples reveals these proteins associated to biological processes governing actin binding, cytoskeletal protein binding, cytoskeletal motor binding, troponin binding, actin filament binding and structural constituent of muscle (Supplementary Fig. S1C). These observations reflect our SWATH-MS analysis of PJ34-treated differentiating lysates (Fig. 4B) and highlight the importance of PARP1-mediated PARylation events that take place on day 1 of differentiation. To better understand how PARylation during early-stage differentiation influences the fully differentiated myotube, we employed Giemsa-Jenner staining of C2C12 myoblasts treated with either PJ34 or BYK204165 during differentiation induction. Cells were fixed on day 6 of differentiation and subjected to an unbiased method of quantification of myogenic differentiation [15]. This analysis revealed subsequently reduced fusion index of myotubes treated with PJ34 (20.15 ± 6.41 S.D) and BYK204165 (24.94 ± 3.21 S.D) (Fig. 5D). These suggest PAR elevation at early-stage differentiation impacts myocyte fusion and PARylation targets during early-stage differentiation are critical to the proper formation of the muscle sarcomere, impacting the overall number of contractile units per fiber.

### Reduction of PARP1 impacts the myoblast transcriptome

**Fig. 6:**
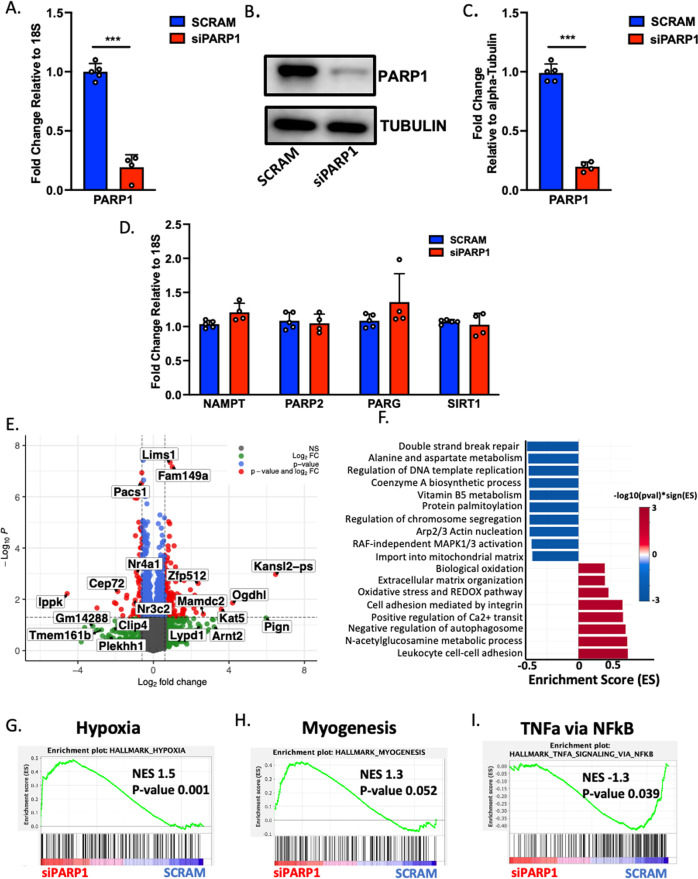
PARP1 transcriptome in myoblasts. **A** qPCR of PARP1 in scrambled sequence controls (*n* = 5) and siRNA PARP1 transfected (*siPARP1*) C2C12 myoblasts (*n* = 4) ***P < 0.001. **B** Western immunoblotting of protein lysates collected from scrambled and *siPARP1* C2C12 myoblasts (*n* = 5). **C** Quantification of scrambled and *siPARP1* transfections by western blotting (*n* = 5). **D** qPCR of Scrambled control and *siPARP1* cDNA for PARP1, PARP2/PARP2, PARG, NAMPT, and SIRT1 transcripts. Scrambled (*n* = 5) and *siPARP1* (*n* = 4). **E** Representative volcano plot of differential gene expression following RNAseq of scrambled and *siPARP1* C2C12 myoblasts. **F** Gene set enrichment analysis (GSEA) of pathways over and under-represented in RNAseq data of *siPARP1* C2C12 myoblasts. **G** Enrichment plots of GSEA in *siPARP1* C2C12 myoblasts for hypoxia, **H** myogenesis and, **I** TNFα via NF-κB.

The muscle differentiation program is transcriptionally regulated by myogenic regulatory factors, some of which have been shown to be influenced by PARP1 [4]. However, the broader elements of PARP1 roles in the muscle transcriptome remain elusive. To provide insight, we conducted RNAseq on undifferentiated C2C12 myoblasts transfected with siRNA-mediated knockdown of *PARP1* (*siPARP1*) (Fig. 6A–C). As PARP1 is a major NAD^+^ consuming enzyme, accounting for up to 90% of cellular PARylation [16], we examined by qPCR, impacts of *siPARP1* on the expression of *PARP2*, *PARG*, *NAMPT,* and *SIRT1*, genes involved in NAD^+^ dependent homeostasis (Fig. 6D). No significant changes were observed in the expression of these genes. Additionally, RNAseq of *siPARP1* myoblasts showed the glucocorticoid binding partner *NR3C2* gene which codes for the mineralocorticoid receptor (MR), as being significantly downregulated (Fig. 6E and Supplementary Table S4). Gene Set Enrichment Analysis of the *siPARP1* differentially expressed genes revealed that consistent with PARP1 established DNA damage repair response functions, the double-strand break repair process was the major deregulated process (Fig. 6E, F). Also suppressed, were pathways and metabolic processes regulating fundamental metabolism including pantothenate metabolism, palmitoylation, and mitochondrial import. Gene Set Enrichment Analysis also reported shifts in major pathways of hypoxia regulation (Normalized Enrichment Score (NES) 1.5, Pvalue 0.001), myogenesis (NES 1.3, *P* value 0.052), and TNF-α via NF-κB (NES −1.3, *P* value 0.039). *PARP1* regulation of hypoxia, as is interaction with the *NF-κB* immune signaling cascades, has been documented [17, 18]. Moreover, these features are reported as being regulated by glucocorticoid signaling [19].

### Reduced PARP1 impacts the glucocorticoid transcriptional response

**Fig. 7:**
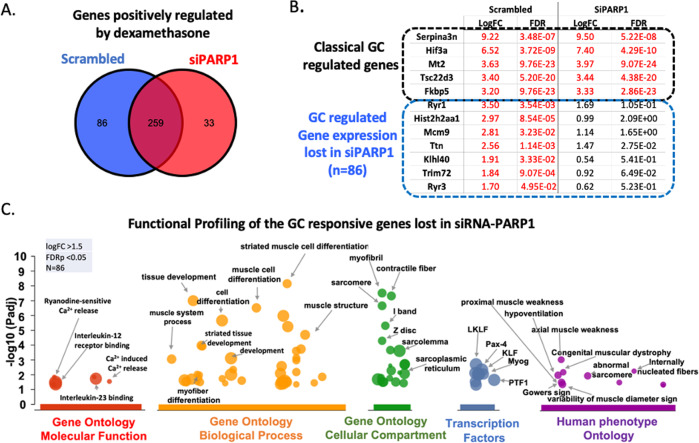
PARP1 partially governs glucocorticoid-mediated transcriptional response. **A** Venn diagram of genes positively regulated by glucocorticoids in Scrambled and *siPARP1* C2C12 myoblasts (>1.5 Fold Change, <0.05 FDR) (*n* = 5 per treatment). **B** Selected list of genes responsive to dexamethasone and gene expression lost in *siPARP1* C2C12 myoblasts. **C** Manhattan plot produced following g:GOSt analysis using the 86 genes whose response to dexamethasone is lost in *siPARP1* C2C12 myoblasts. The plot shows overrepresented processes by Molecular function (red), Biological process (orange), Cellular compartment (green), Transcription factors (blue), and Human phenotype (purple).

Finally, as we show PARylation as being a glucocorticoid-sensitive post-translational modification (Fig. 2D, E), as well as *NR3C2* (MR) being downregulated in *siPARP1* C2C12 myoblasts (Fig. 6E), and glucocorticoid signaling being a determinant of muscle turnover [19–22], we set out to test if *PARP1* influences the glucocorticoid activated transcriptional program. To do this, we treated *siPARP1* and scrambled transfected C2C12 myoblasts with dexamethasone for 24 hours. RNA was collected and sequenced for differential gene expression. We found that *siPARP1* myoblasts retained the ability to upregulate genes in response to dexamethasone, sharing activation of typical glucocorticoid-regulated genes including *SerpinA3N* (9.50 fold change; 5.22 × 10^−08^ False Discovery Rate (FDR), *HIF3α* (7.40 fold change; 4.29 × 10^−10^ FDR) and *Mt2* (3.97 fold change; 9.07 × 10^−24^ FDR). However, we observed that the expression of 86 glucocorticoid-induced genes was lost in dexamethasone-treated *siPARP1* myoblasts (Fig. 7A, B and Supplementary Table S5). A tabular selection of these is presented for scrambled and *siPARP1* myoblasts alongside classically glucocorticoid-activated genes (Fig. 7B), and the full list is provided (Supplementary Table S5). This cohort of genes, differentially regulated by dexamethasone in scrambled controls but not *siPARP1* myoblasts, were subjected to functional profiling using g:Profiler [23]. This revealed enrichment for gene ontological processes and functions of significance to skeletal muscle physiology including calcium release (Padj. 1.49 × 10^−2^), as well as biological processes including straited muscle differentiation (Padj. 1.03 × 10^−8^) and cellular development (Padj. 3.34 × 10^−6^) (Fig. 7C). Furthermore, enrichment of the cellular compartment forming the thin filament of the myofibril was recorded (I band (FDR 5.06 × 10^−6^); Z disc (FDR 5.59 × 10^−5^)). Mapping of these 86 genes for transcription factor motifs generated enrichment for key regulatory factors of muscle including *Pax4*, a regulator of muscle protein turnover [24] (Padj. 4.67 × 10^−3^), *LKLF* (Padj. 5.72 × 10^−3^), *PTF1* (Padj. 8.84 × 10^−3^) and *myogenin* (Padj. 8.98 × 10^−3^). Finally, human phenotype mapping identifies this gene cohort as being characteristic of skeletal muscle pathology including proximal muscle weakness, muscular dystrophy and Gower’s sign [25]. These data indicate there are a subset of genes regulated by glucocorticoids in skeletal muscle which are also dependent on *PARP1*.

## Discussion

Herein, we demonstrate PARP1 and PARylation as being dynamically regulated during murine and human skeletal muscle differentiation. Specifically, we detail a conserved early-stage PAR accumulation that occurs within 24 h of myogenic induction that is both active and metabolically sensitive. We also show PARP1 may have roles in the cellular response to glucocorticoids, via regulation of a cohort of genes required for functional skeletal muscle.

### PARP1 and PARylation are dynamic during skeletal muscle myogenesis

Suppression of PARP1 elicits beneficial metabolic effects which include enhanced exercise performance, increased energy expenditure, enhanced mitochondrial function, and resistance to oxidative stress [7–9, 26]. Additionally, PARP1 and PAR levels are dynamically and differentially regulated in response to exercise between aged untrained and aged trained muscle [27]. Our results establish PAR deposition by PARP1 as well as its auto-PARylation occurring within the first 24 h of myogenesis, supporting recent work showing PARP1-mediated PAR deposition following MYOD-driven transdifferentiation of fibroblasts into myoblasts [28]. This increased PAR accumulation could be ascribed to metabolic pathway shifts from glycolysis to oxidative phosphorylation as the major energy source during myogenesis, which has been reported in embryonic stem cells and induced pluripotent stem cells [29, 30]. Initiation of myogenesis upregulates the muscle-restricted gene NMRK2, and this switch increases NAD^+^ biosynthesis to drive PARP1 activity [31]. While the molecular recipients of the differentiating day 1 PAR accumulation are yet to be identified, existing evidence indicates MYOD is a likely target. Our data also show that this PAR accumulation is sensitive to changes in metabolism. Notably, we observed higher PAR levels in glucose-deprived conditions during myogenesis. However, PAR levels are induced in conditions of high glucose levels and fed states [7, 32]. These energetic requirements of day 1 differentiating myoblasts are likely specific to this point of myogenesis. This similarly has been found with autophagy and mitochondrial biogenesis which also upregulate PAR on day 1 [33, 34]. We also demonstrate that within myogenesis, that the troponins are also governed by PARP1 inhibition. It is therefore probable that PARP1, either through the direct or enzymatic application of PAR chains, coregulates their mRNA transcription. Incidentally, impacts over troponin expression by PARP1 hold relevance for cardiac muscle [35]. Given the nature of PARP1 PARylating activity, we also showed NAD^+^ precursor supplementation did not significantly impact PAR dynamics. This is possibly due to buffering mechanisms in response to NAD^+^ availability [36], the specific inhibition of PARP1 was successful in reducing PAR supported via observed increases in NAD^+^ (Supplementary Fig. 1A).

### PARP1-mediated PARylation contributes to myogenesis

We postulated that PARP1-mediated PAR accumulation occurring on day 1 of myogenesis is to an extent, not dependent on myotube formation, but rather exerts consequences for the developed myotube. Using the broad-spectrum PARP inhibitor PJ34 and the PARP1-specific inhibitor BYK204165, we show that the expression profile changes are indeed related to PARP1-mediated PARylation on the first day of myogenesis. We also found downregulation of *PARP1* alone in undifferentiated C2C12 myoblasts was sufficient to cause shifts in the muscle transcriptome, and subsequent biological processes comparable to those of early-stage PARP1 inhibition during myogenesis. Further insight into which substrates are PARylated within the first day of myogenesis where PAR disposition is at its highest will be useful in defining downstream signaling. In this regard, nuclear PARP1-mediated PARylating activities are increased in the myoblast following successful transdifferentiation from fibroblasts, providing merit for this [28]. It is, however, likely that site-specific PARylation of key myogenic regulators occurs, although this remains to be tested, once performed, it would reveal specific amino acid sequences serving to impact the myogenic protein activity and the continual process of muscle turnover. With regards to skeletal muscle, the myogenic transcriptional regulator Yin Yang1 (*YY1)* has been directly shown to be both a recipient of PARylation by PARP1 [37], and regulated by the immunomodulatory NF-kB pathway during myogenesis [38]. Crosstalk between PARP1 and NF-kB has been reported in several cell and tissue types [17, 39, 40]. Therefore, it is probable that a greater level of coordination between the NF-kB pathway and PARylation exists and remains to be explored in skeletal muscle. Another direct recipient of PARylation and regulator of myogenic progression is the transcription factor CCAAT/enhancer binding protein beta (C/EBPb), which has been demonstrated to be PARylated during adipogenesis [2, 3]. Given C/EBPb roles in satellite cells, the myocyte, and its interactions with MYOD [41–43], there is potential for C/EBPb PARylation in muscle tissue. Additional mechanisms of both PARP1 and catalytic PAR control are also not discounted and exert influence over 3D chromatin organization [44, 45]. However, it should be noted that PARP2 which also contributes to PAR accumulation, has demonstrated roles in myogenesis and skeletal muscle structure [46]. Collectively, our presented results suggest that both PARP1 and its PARylating activity hold influence over the skeletal muscle phenotype, and further underscores PARP1’s role as a multifaceted protein being able to bind to nucleic acids as well catalyzing the PARylation of target substrates [1, 47, 48].

### PARP1 exerts influence over skeletal muscle glucocorticoid transcriptional response

Evidence for PARP1 in regulation of GR-mediated transcriptional response has been presented in other cells [49]. Furthermore, *PARP1* null mice have increased cortisol levels [50]. Our transcriptomic analysis of *siPARP1* C2C12 myoblasts reveals differentially expressed changes in the *N3RC2* gene coding for MR, and while not as ubiquitous as its GR counterpart, MR can also bind glucocorticoids with higher affinities [19]. Moreover, we demonstrate pathways commonly classified as glucocorticoid controlled, including hypoxia, myogenesis, and TNF-α response through NF-κB, being shifted in *siPARP1* myoblasts (Fig. 6g, h, i). Because PARP1 has documented roles in the NF-κB pathway for control of inflammatory response [17] and the mechanism of anti-inflammatory effects of glucocorticoids involves the repression of NF-κB, it is plausible that this glucocorticoid-mediated effect occurs via PARP1. Furthermore, NF-κB has also been implicated in differentiation programs of other tissues, including myogenesis [38, 39, 51]. In this regard, PARP1 and its PARylating activity exert influence over osteoclast differentiation and bone remodeling via *NF-κB* dependent transcription of *IL-1β* [39], and glucocorticoid-induced osteoporosis is a consequence of the inhibition of IL-1 production [52]. Furthermore, as with PARP1, the suppression of NF-κB is fundamental for driving myogenesis [38, 51], while dexamethasone treatment at the myoblast stage enhances myogenesis [20–22]. Concurrently, we demonstrate that application of dexamethasone during differentiation induction of myoblasts reduces PAR levels (Fig. 2D) indicating glucocorticoids have impacts on PARP1-mediated PARylating activity. Recent studies have demonstrated that the tumor susceptibility gene TSG101 binds to and enhances GR transcriptional activity [53] and similarly, PARP1 drives enzymatic activity for DNA damage-induced IKK-NF-κB activation [54]. Therefore, we speculate a similar paradigm in which glucocorticoids can regulate PARP1-mediated PARylation within the skeletal muscle via PARP1 interacting partners. Together with our findings, there is suggested the potential for PARP1 and GR interactions for influencing glucocorticoid-mediated outcomes and muscle turnover.

By examining the transcriptomic changes in *siPARP1* C2C12 myoblasts treated with dexamethasone, we observe that the expression of glucocorticoid canonical target genes such as *FKBP5* remained intact (Fig. 7B). We observed differential expression in a cohort of genes whose response following dexamethasone treatment was lost in *siPARP1* myoblasts. These imply that PARP1 exerts influence over glucocorticoid transcriptional response in skeletal muscle. Furthermore, PARP1 has demonstrated involvement in myopathy [55], as does chronic glucocorticoid exposure [21, 56]. These indicate potential for PARP1 roles in the transcriptional pathways manifesting glucocorticoid-induced myopathy, necessitating broader studies. PARP1 mediates the glucocorticoid responsiveness of Gonadotropin-Releasing Hormone receptors [57], and here our findings demonstrate aspects of glucocorticoid-mediated impacts on skeletal muscle may occur through PARP1. However, it should also be appreciated that differential expression of glucocorticoid response genes could potentially be regulated by downstream events who themselves are impacted by *siPARP1*. Further investigation into direct GR and PARP1 interactions within the skeletal muscle would therefore establish clearer links into the extent PARP1 holds over overall glucocorticoid response.

**Fig. 8:**
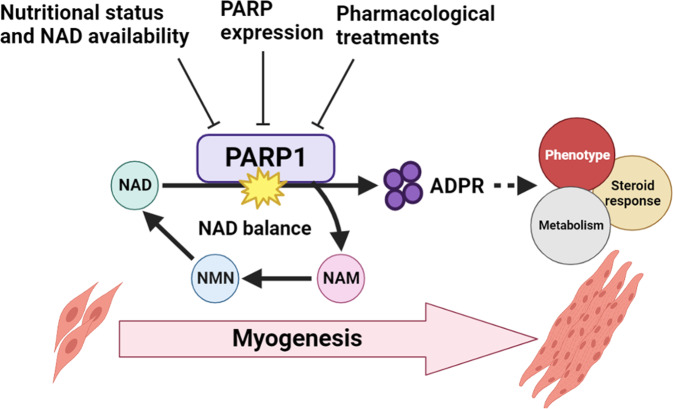
PARP1-driven PARylation targets during early-stage differentiation and is central to proper development of the contractile unit. PARP1 in myoblasts also influences transcriptional regulation of glucocorticoids. These actions highlight the importance of NAD+ availability and PARylation to skeletal muscle.

Collectively, our studies demonstrate further potential for PARylation actions in skeletal muscle physiology and response to glucocorticoids (Fig. 8). While this might provide novel avenues for PARP inhibition in amelioration of chronic glucocorticoid-induced side effects, potential negative impacts over skeletal muscle turnover and repair should be considered.

## Materials and methods

### Cell culture

The murine muscle myoblast cell line C2C12 was purchased (ATCC, VA, USA) and maintained in proliferation media, composed of Dulbecco’s Modified Eagle’s Medium (DMEM) 25 mM glucose (Lonza, UK) supplemented with 10% (v/v) fetal bovine serum (FBS) (Thermo, UK) and 1% Penicillin/Streptomycin (P/S) (Thermo, UK). Upon cells reaching 70–80% confluence, the differentiation medium, composed of DMEM 25 mM glucose supplemented with 2% horse serum (HS) (Thermo, UK) and 1% P/S, was added to induce differentiation. The myoblasts were allowed to differentiate for 6 days with fresh differentiation medium added every other day, sufficient for the successful development of mature myotubes. The human muscle myoblast cell line LHCN-M2 (Evercyte, Germany) was grown in proliferation media as described [58], composed of a 4:1 ratio of DMEM and Medium 199 (Sigma-Aldrich, UK) supplemented with 15% FBS, 200 mM HEPES (Thermo, UK), 0.03 µg/ml zinc sulfate (Sigma-Aldrich, UK), 1.4 µg/ml vitamin B12 (Sigma-Aldrich, UK), 0.055 µg/ml dexamethasone (Sigma-Aldrich, UK), 2.5 ng/ml hepatocyte growth factor (Proteintech, UK), 10 ng/ml basic fibroblast growth factor (Sigma-Aldrich, UK) and 1% P/S. The differentiation media of LHCN-M2 was composed of a 4:1 ratio of DMEM and Medium 199 supplemented with 2% HS and 1% P/S. LHCN-M2 differentiation was performed similarly to C2C12 myoblasts.

### Cell treatments

Myoblasts were treated with either vehicle controls or the PARP inhibitors BYK204165 (Tocris, UK) and PJ34 (MedChemExpress, NJ, USA) at working concentrations of 10 µM, as well as Rucaparib (Selleck Chemical) treatment at a working concentration of 1 µM. Treatments with nicotinamide riboside (NR) (Chromadex, CA, USA) were performed at 0.5 mM, FK866 (Sigma-Aldrich, UK) at 50 nM, and dexamethasone at 1 µM. All treatments were diluted in the differentiation medium and performed during differentiation induction of myoblasts, after which treatments were washed out after 1 day (24 h) of differentiation and replaced with fresh differentiation medium, and myoblasts were left to differentiate as described.

### Transfection of silencing RNA

Cells were seeded at an approximate density of 100,000 cells/well in six-well cell culture plates and incubated for at least 3 h to allow adherence. The siRNA transfection mix was composed of Lipofectamine 2000 (Thermo, UK) and 0.5 µg/µl of siRNA PARP1 predesigned from three pooled siRNA PARP1 sequences (Merck, UK) or scrambled siRNA control sequence (Merck, UK). Transfection was conducted in Opti-Mem serum and antibiotic-free media (Thermo, UK) and incubated for 24 h, after which the transfection mix was replaced with fresh medium.

### RNA extraction and quantitative PCR

Cells were washed with 1x PBS and harvested in TRIzol Reagent (Life Technologies, UK). Total RNA was isolated using chloroform extraction and isopropanol precipitation. RNA clean-up and purification were conducted using a column purification kit (Zymo, Germany) according to instructions and quantity was measured by Nanodrop. Samples designated for RNAseq were further assessed for quality using the 2100 Bioanalyzer Instrument (Agilent Technologies, UK) with all samples having at least an RNA integrity number of >9.5.

For quantitative PCR (qPCR), reverse transcription was performed on extracted RNA using a High-Capacity cDNA Reverse Transcription Kit (Thermo, UK) according to the manufacturer’s instructions. cDNA was added to TaqMan™ Universal PCR Master Mix (Thermo, UK) with the appropriate primer pairs as follows: *PARP1* forward: CCTGAACAACGCAGACAGC, *PARP1* reverse: CGTTGTGCGTGGTAGCATGA, *PARP2* forward: GGAAGGCGAGTGCTAAATGAA, *PARP2* reverse: GGAAGGCGAGTGCTAAATGAA, *NAMPT* forward: CGCCATCTCCTTGAATGA, *NAMPT* reverse: GCACCACTAATCATCAGACC, *PARG* forward: GTGCCAGTTTCGATCCGTAGA, *PARG* reverse GGCCAGCATCGTGTAGATGA, *SIRT1* forward: GGCTACCGAGACAACCTCCTG, *SIRT1* reverse: AGTCCAGTCACTAGAGCTGGCG. Reactions were performed in 384 well plates and conducted using QuantStudio™ 7 Flex Real-Time PCR System (Applied Biosystems, UK) in single-plex format. All reactions were normalized to 18S rRNA (VIC^TM^) (Applied Biosystems, UK). Data were collected as Ct values and used to obtain ΔΔCt values, and subsequently expressed as fold change ± standard error of the mean (S.D).

### RNAseq

Library preparation and sequencing were conducted by Novogene Inc., UK, original fastq files can be found on EBI Array Express E-MTAB-12343. Reads were mapped using the kallisto RNAseq quantification program [59]. Analyses were carried out using R in RStudio and Bioconductor. Briefly, transcript quantification data were summarized to genes by tximport and normalized using edgeR. Normalized and filtered data were variance stabilized with voom function from limma and differentially expressed genes were identified with limma. Functional enrichment of differential expressed genes was conducted using g:Profiler [60].

### Cell lysis and western blotting

For whole-cell lysis, cells were first washed in ice-cold 1x PBS and subsequently scraped in RIPA buffer (50 mM Tris pH 8.0, 150 mM NaCl, 0.5% (w/v) sodium deoxycholate, 0.1% (w/v) SDS, 1% NP-40) supplemented with 1× Pierce™ EDTA-free protease inhibitor cocktail (Thermo, UK) and incubated on ice for 30 min. Lysates were clarified by centrifugation at 12,000 rpm for 15 min at 4^o^C. The supernatant was recovered and stored at -80^o^C until use. Total protein concentration was determined by detergent-compatible protein assay (Bio-Rad, UK) according to the kit’s instructions.

Total proteins were loaded and resolved on fixed percentage acrylamide SDS-PAGE gels and subsequently transferred onto PVDF membranes using Trans-Blot Turbo Transfer System (Bio-Rad, UK). Membranes were blocked with 5% bovine serum albumin (BSA) diluted in 1x TBS-T and incubated with primary antibodies overnight at 4^o^C: PARP1 (39559, Active-Motif, Belgium), PAR (MABE1031, Merck Millipore, UK), MYOD (sc-377460, Santa Cruz Biotechnology, Inc., USA), Hexokinase II (ab227198, Abcam, UK), Myogenin (ab1835, Abcam, UK), NF-kB (8242, Cell Signalling Technology, UK), α-Tubulin (sc-5286, Santa Cruz Biotechnology, Inc., USA), and subsequently with HRP-conjugated anti-mouse or anti-rabbit secondary antibodies (Dako, Denmark) for 1 h at room temperature. Blots were developed with Pierce™ ECL Western Blotting Substrate (Thermo, UK) and visualized using the G:BOX Chemi XX6 system (Syngene, UK). Bands were measured using Image J densitometry and normalized to those of loading controls.

### Immunofluorescence

Cells grown on coverslips were rinsed with 1× PBS and fixed with 4% PFA (Thermo, UK). Permeabilization was performed with 0.1% Triton X-100 (Sigma-Aldrich, UK) and blocked with 10% goat serum (Life Technologies, UK). Coverslips were incubated with primary antibodies overnight at 4 ^o^C, before incubation with secondary antibodies and DAPI nuclear dye (Thermo, UK) for 1 h at room temperature. Coverslips were mounted on slides and allowed to set prior to imaging.

### Unbiased proteomics

Unbiased mass spectrometry was carried out on a SCIEX TripleTOF 6600 instrument with samples analyzed in both SWATH (Data Independent Acquisition) and IDA (Information Dependent Acquisition) modes for quantitation and spectral library generation respectively. IDA data were searched together using ProteinPilot 5.0.2 to generate a spectral library and SWATH data was analyzed using Sciex OneOmics software extracted against the locally generated library. Log fold change and statistical analysis were calculated as described [60].

### NAD^+^ measurement

NAD^+^ was measured in lysates extracted in 0.6 N perchloric acid as described [61]. Briefly, standards or samples in phosphate buffer were combined with the cycling mixture composed of 2% ethanol, 100 µg/ml alcohol dehydrogenase, 10 µg/ml diaphorase, 20 µM resazurin, 10 µM flavin mononucleotide, 10 mM nicotinamide and 0.1% BSA, in 100 mM phosphate buffer pH 8.0. Resorufin accumulation was evaluated by reading excitation at 544 nm and emission at 590 nm.

### Giemsa–Jenner staining of myotubes

Cells stained during differentiation time points were performed as described [15]. Briefly, cells were fixed in methanol and stained with Jenner (Alfa Aesar, UK) diluted in 1 mM sodium phosphate pH 5.6, followed by Giemsa (Alfa Aesar, UK) diluted in 1 mM sodium phosphate pH 5.6. Cells were imaged using a camera attached to an inverted microscope. Protein-rich myotube fibers presented themselves as dark/deep purple while nuclei-stained shallow purple/pink. Total number of nuclei per image was manually scored and the number of nuclei inside myotubes was expressed as a percentage of total nuclei to obtain the fusion index.

### Statistical analysis

Students *t* test or ANOVA statistical comparisons were used with the Graphpad Software Inc. Prism version 9. Western blots were quantified by densitometry using imageJ. Data are presented as mean ± S.D with statistical significance determined as *P < 0.05, **P < 0.01, ***P < 0.001. Differences between two groups were determined using unpaired t-test compared treatments or genotypes. Statistical analysis derived from qPCR data was determined using ΔΔCt values throughout.

## Supplementary information

Supplementary figure 1.

Supplementary figure 2.

Supplementary table 1

Supplementary table 2

Supplementary table 3

Supplementary table 4

Supplementary table 5

Original blots

## Data Availability

The datasets generated for this study are available on request to the corresponding author. RNAseq files are available at Array Express.

## Code availability

Code generated using RStudio version 4.2 and is available upon request.
