# Supplementary figures and images for: PARP1 mediated PARylation contributes to myogenic progression and glucocorticoid transcriptional response
Source: Cell Death Discov. 2023 Apr 22;9:133. doi: 10.1038/s41420-023-01420-2 (PMC10121420; doi:10.1038/s41420-023-01420-2)

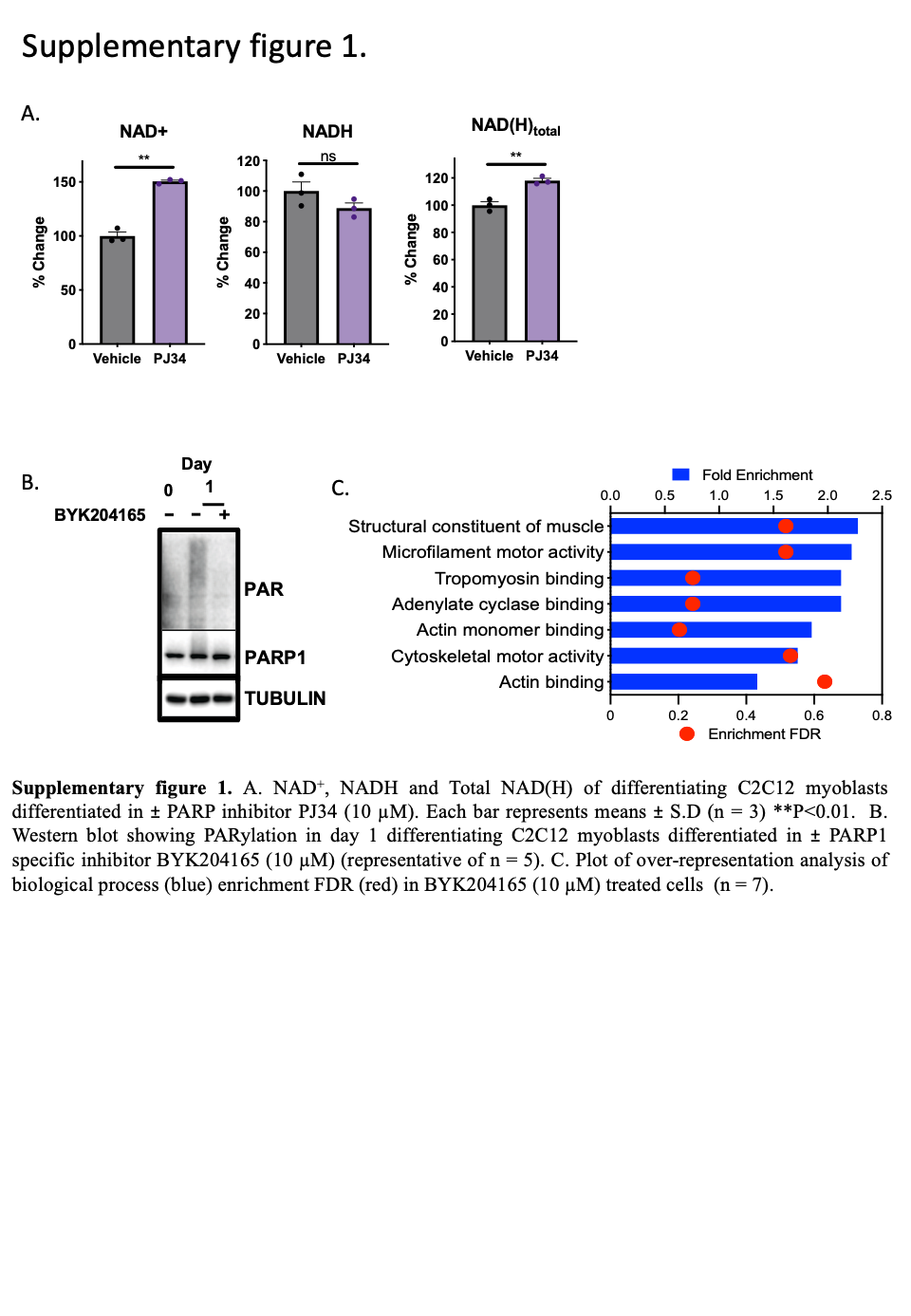

Supplement: Supplementary file 1 — Supplementary figure 1. [file 41420_2023_1420_MOESM1_ESM.tif]

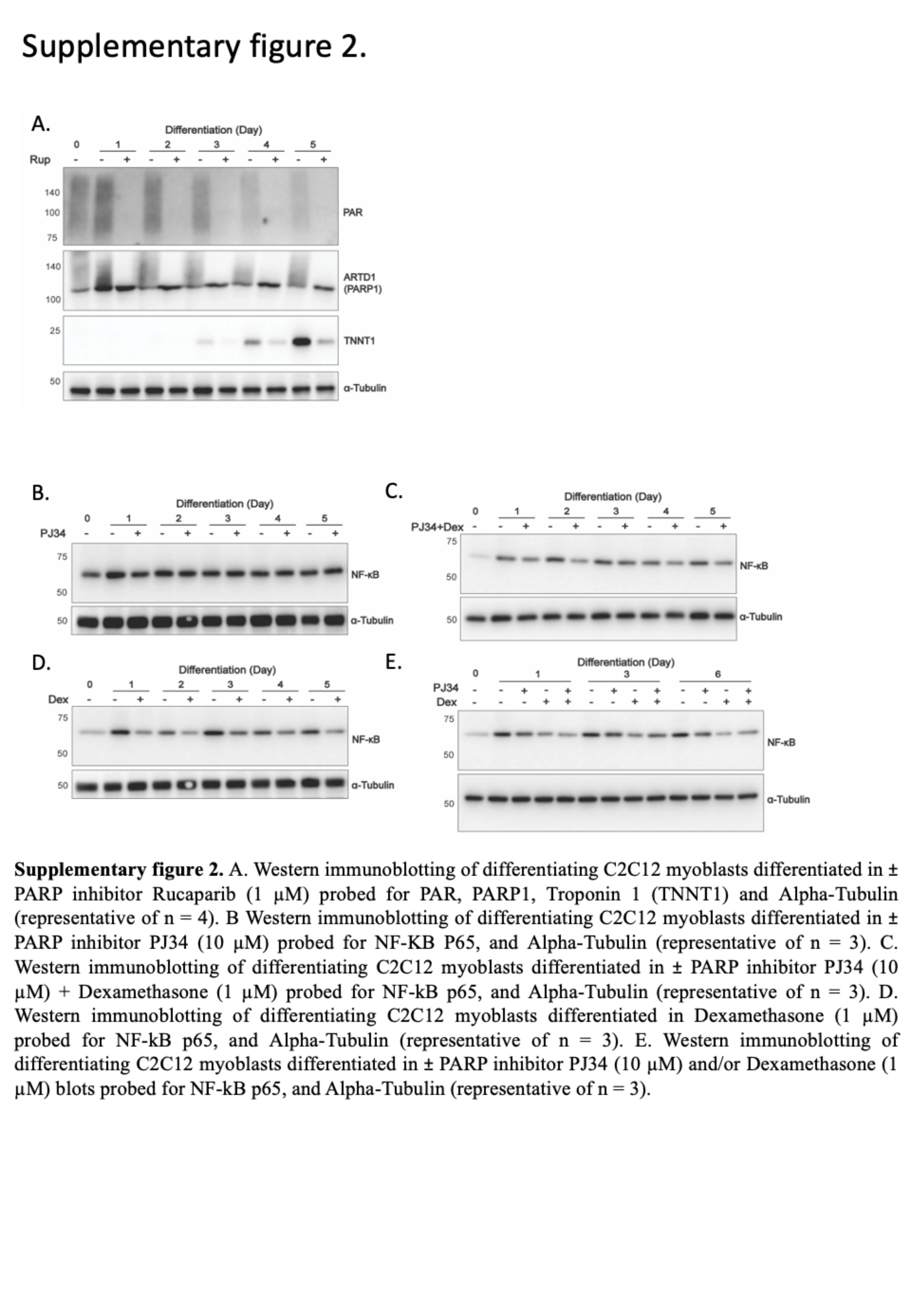

Supplement: Supplementary file 2 — Supplementary figure 2. [file 41420_2023_1420_MOESM2_ESM.tif]
