# Supplementary material for: PARP1 mediated PARylation contributes to myogenic progression and glucocorticoid transcriptional response
Source: Cell Death Discov. 2023 Apr 22;9:133. doi: 10.1038/s41420-023-01420-2 (PMC10121420; doi:10.1038/s41420-023-01420-2)
Supplement: Supplementary file 8 — Original blots [file 41420_2023_1420_MOESM8_ESM.pptx]

## Slide 1
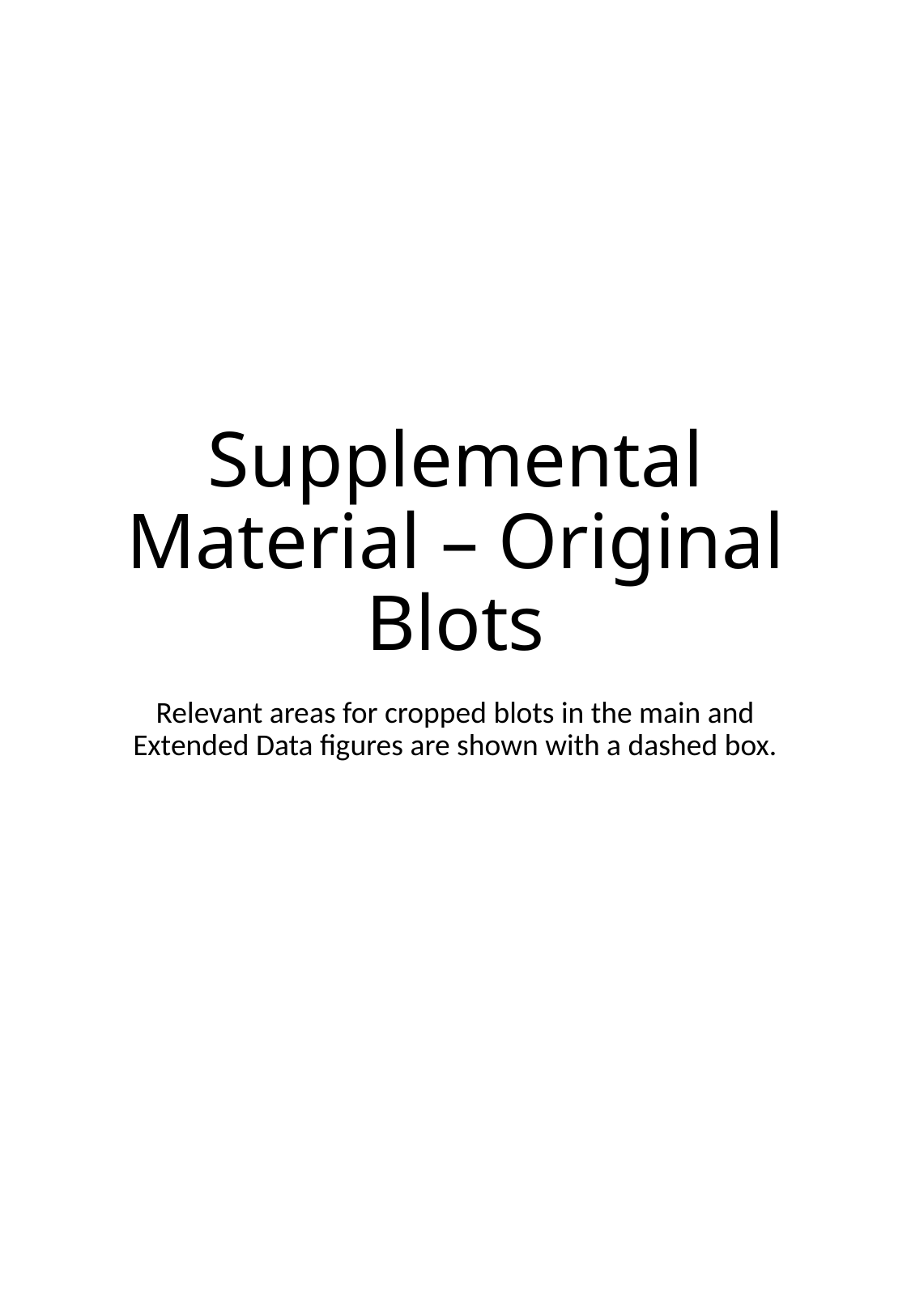

# Supplemental Material – Original Blots
Relevant areas for cropped blots in the main and Extended Data figures are shown with a dashed box.

## Slide 2
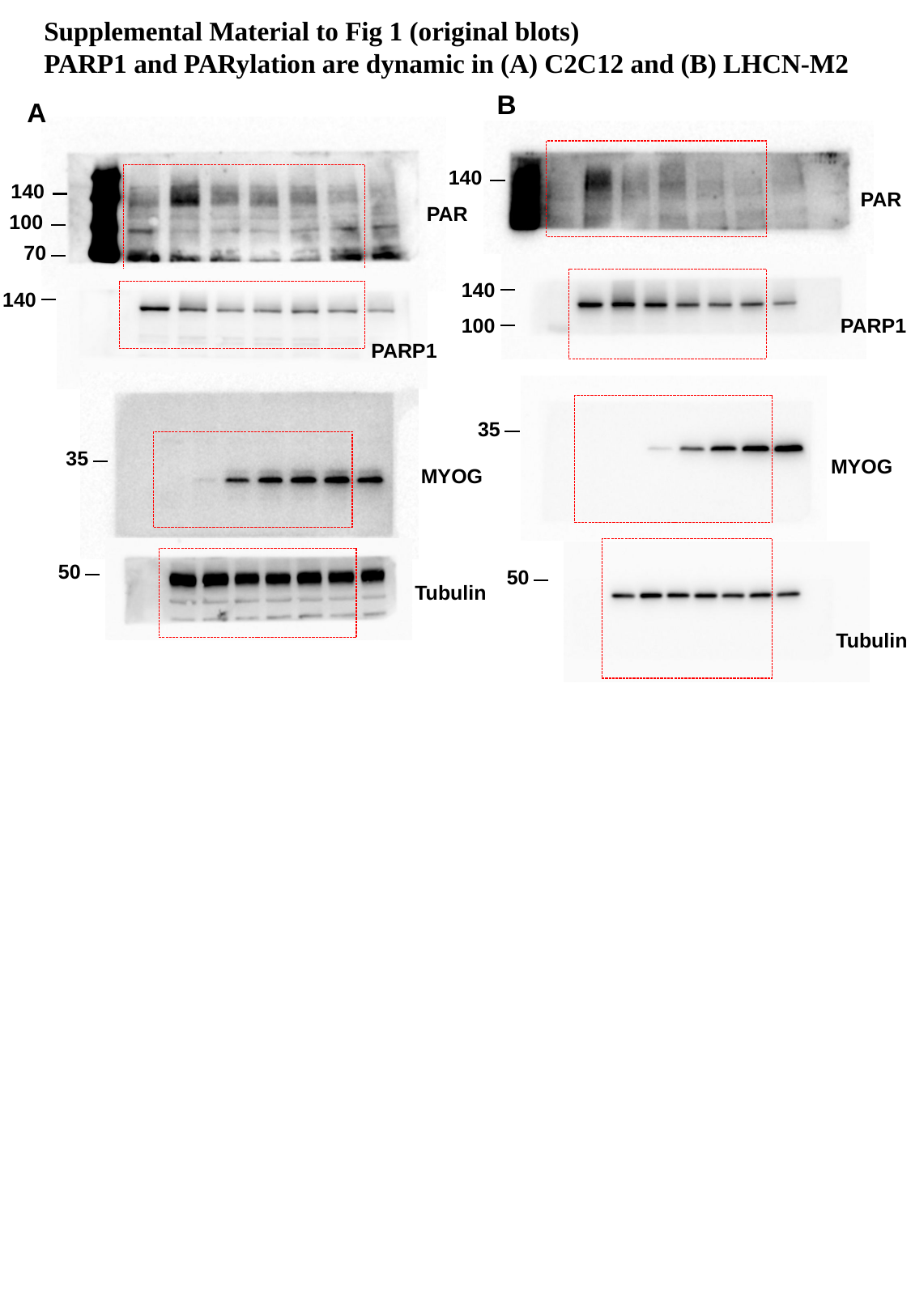

Supplemental Material to Fig 1 (original blots)
PARP1 and PARylation are dynamic in (A) C2C12 and (B) LHCN-M2
B
A
140
140
PAR
PAR
120
100
70
140
140
PARP1
100
PARP1
35
35
MYOG
MYOG
50
50
Tubulin
Tubulin

## Slide 3
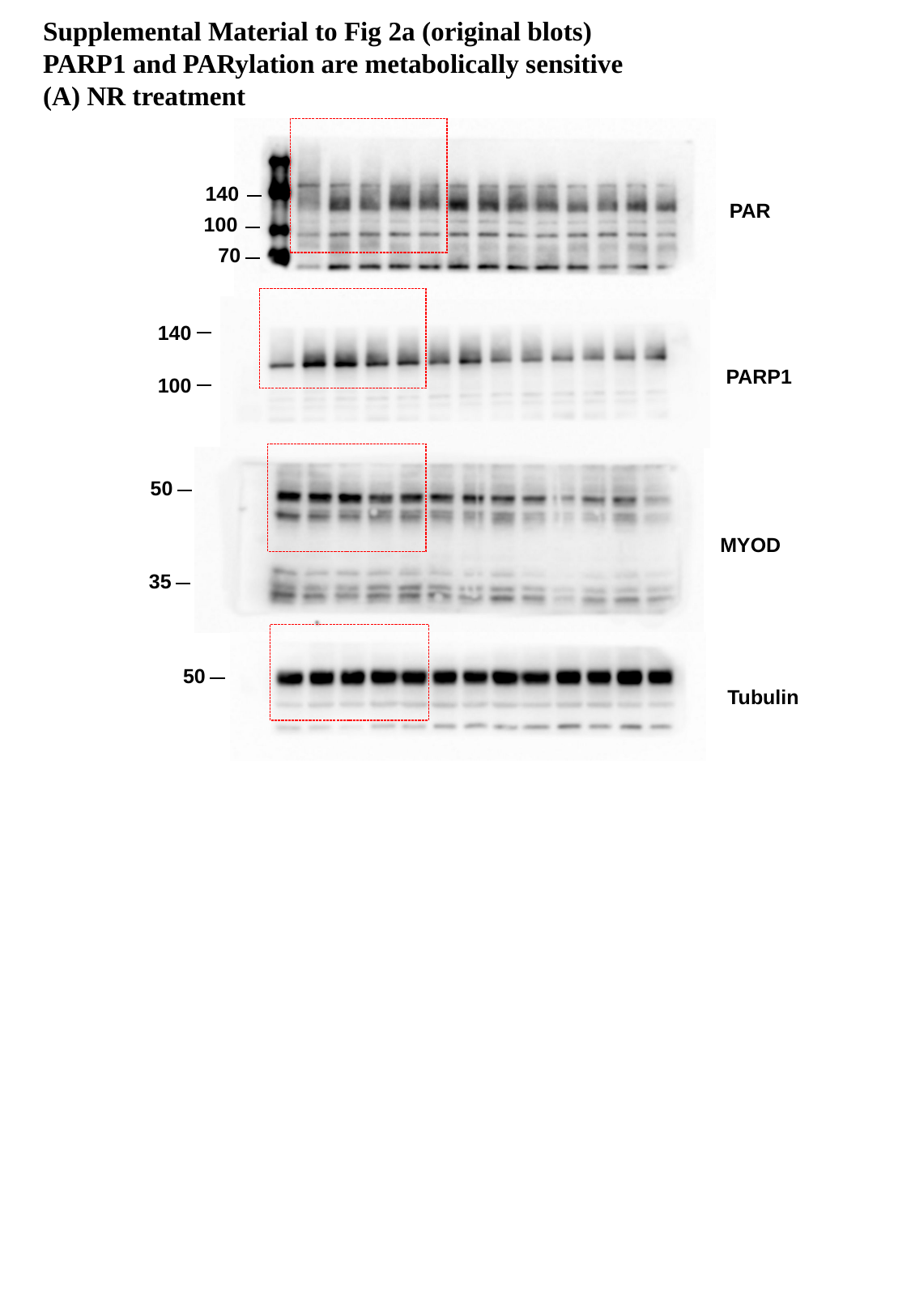

Supplemental Material to Fig 2a (original blots)
PARP1 and PARylation are metabolically sensitive
(A) NR treatment
140
PAR
100
70
140
PARP1
100
50
MYOD
35
50
Tubulin

## Slide 4
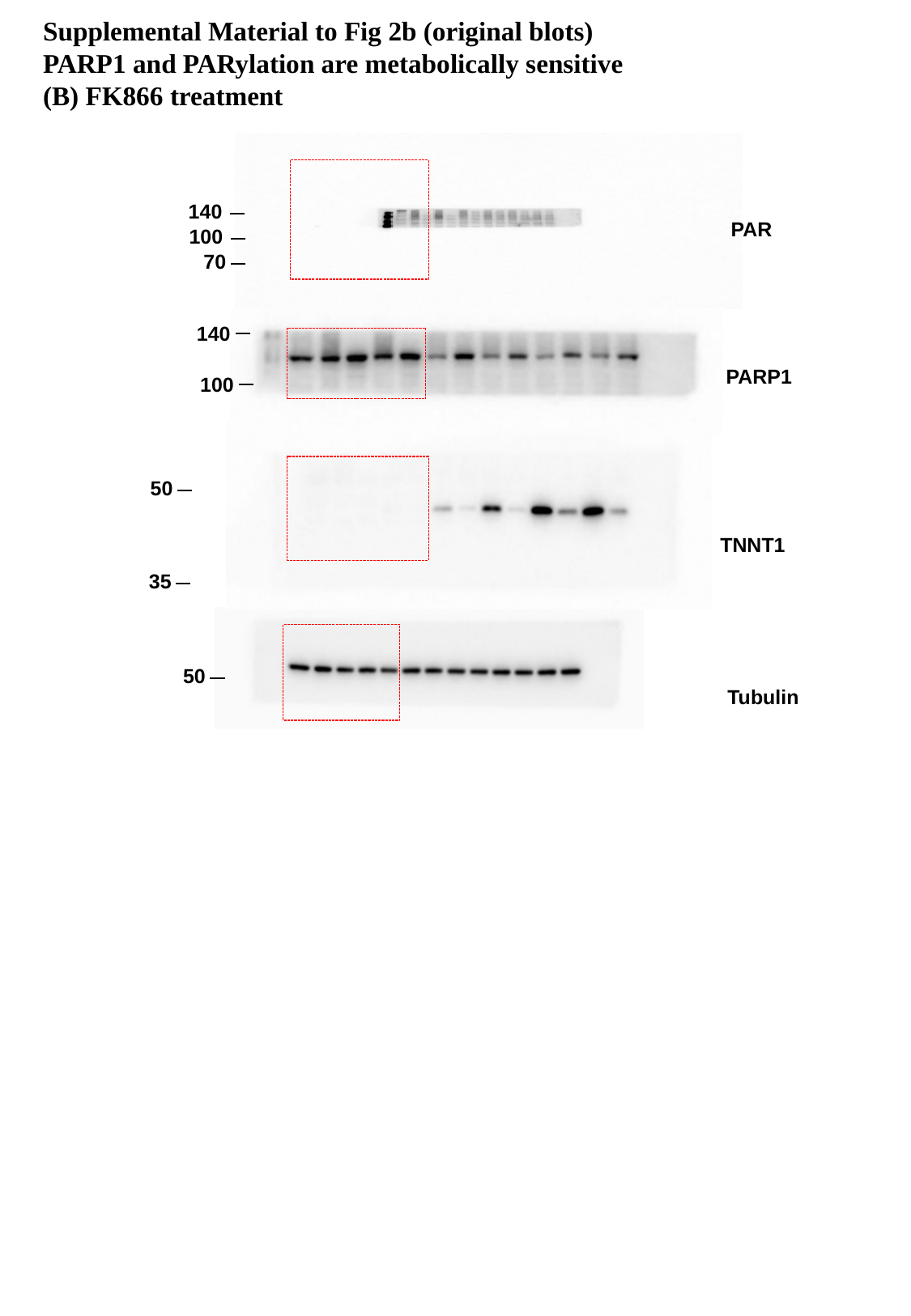

Supplemental Material to Fig 2b (original blots)
PARP1 and PARylation are metabolically sensitive
(B) FK866 treatment
140
PAR
100
70
140
PARP1
100
50
TNNT1
35
50
Tubulin

## Slide 5
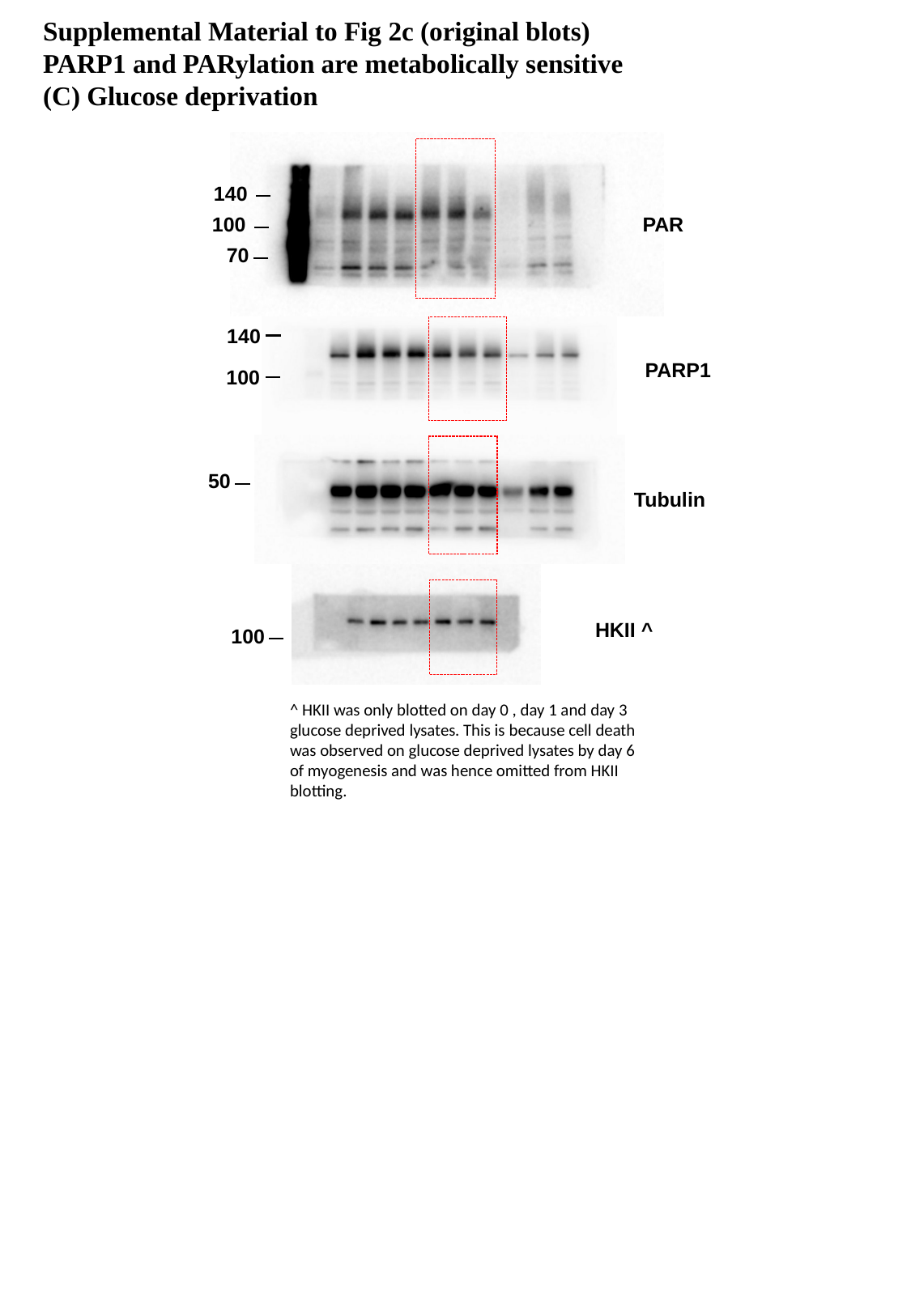

Supplemental Material to Fig 2c (original blots)
PARP1 and PARylation are metabolically sensitive
(C) Glucose deprivation
140
PAR
100
70
140
PARP1
100
50
Tubulin
HKII ^
100
^ HKII was only blotted on day 0 , day 1 and day 3 glucose deprived lysates. This is because cell death was observed on glucose deprived lysates by day 6 of myogenesis and was hence omitted from HKII blotting.

## Slide 6
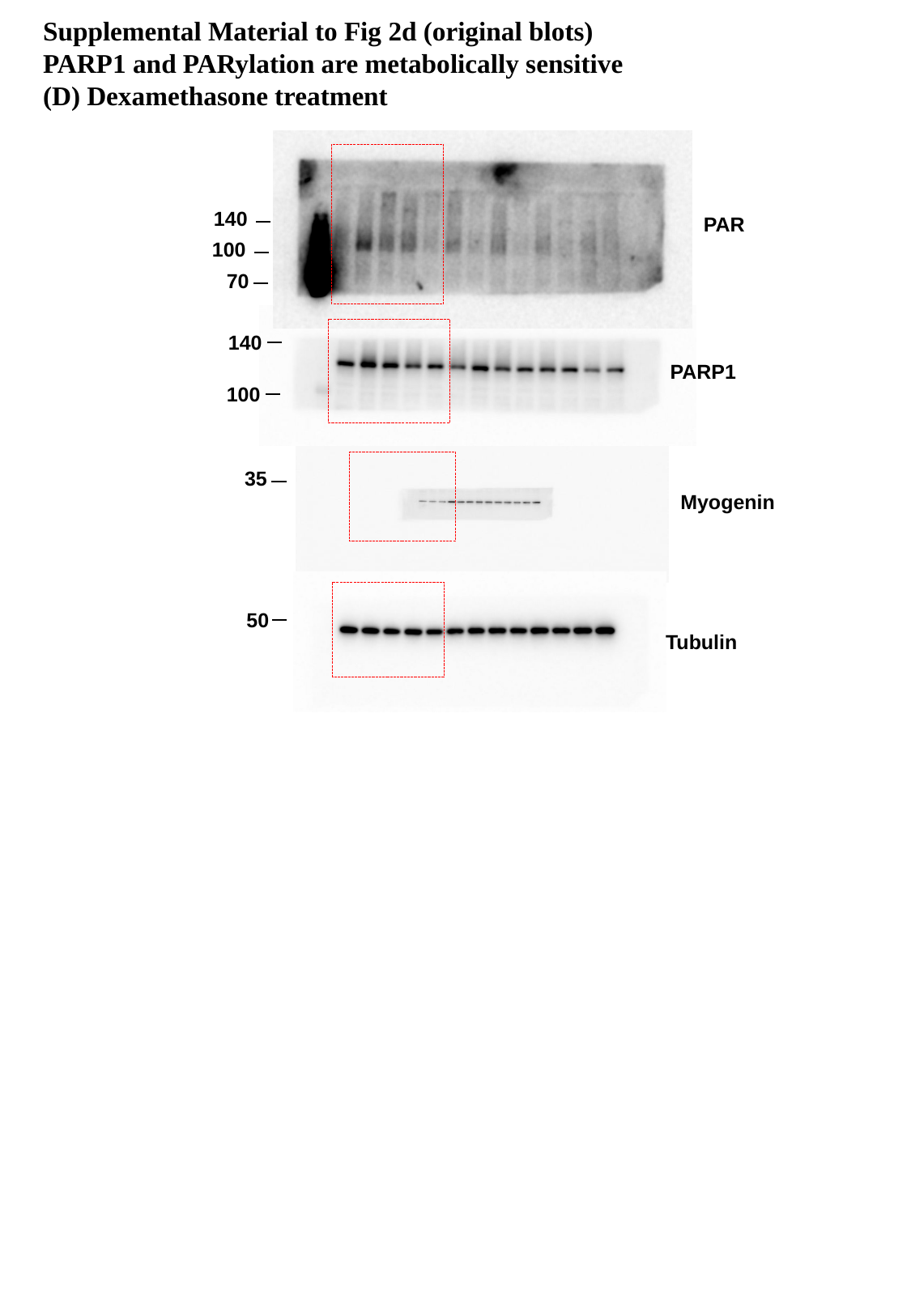

Supplemental Material to Fig 2d (original blots)
PARP1 and PARylation are metabolically sensitive
(D) Dexamethasone treatment
140
PAR
100
70
140
PARP1
100
35
Myogenin
50
Tubulin

## Slide 7
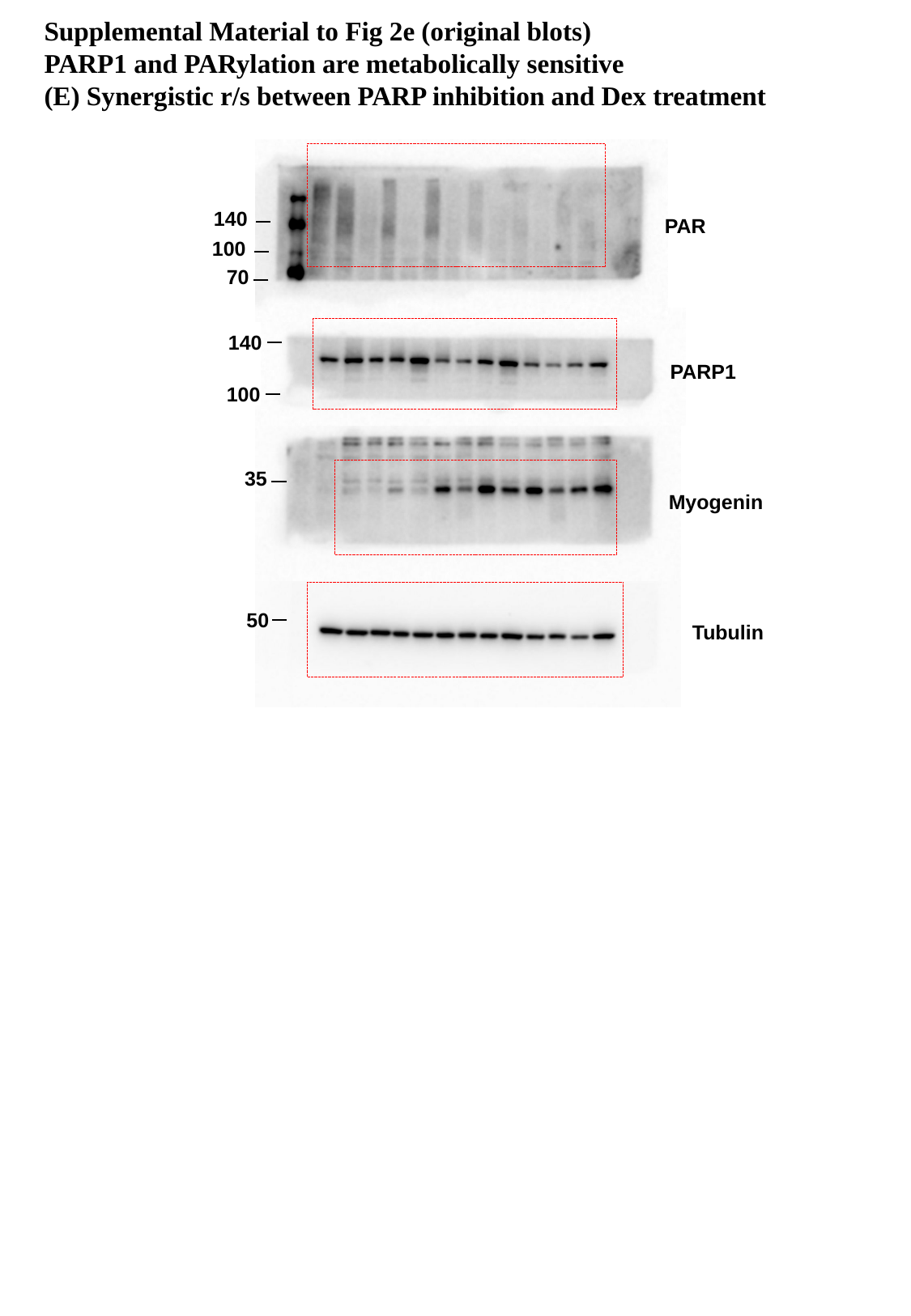

Supplemental Material to Fig 2e (original blots)
PARP1 and PARylation are metabolically sensitive
(E) Synergistic r/s between PARP inhibition and Dex treatment
140
PAR
100
70
140
PARP1
100
35
Myogenin
50
Tubulin

## Slide 8
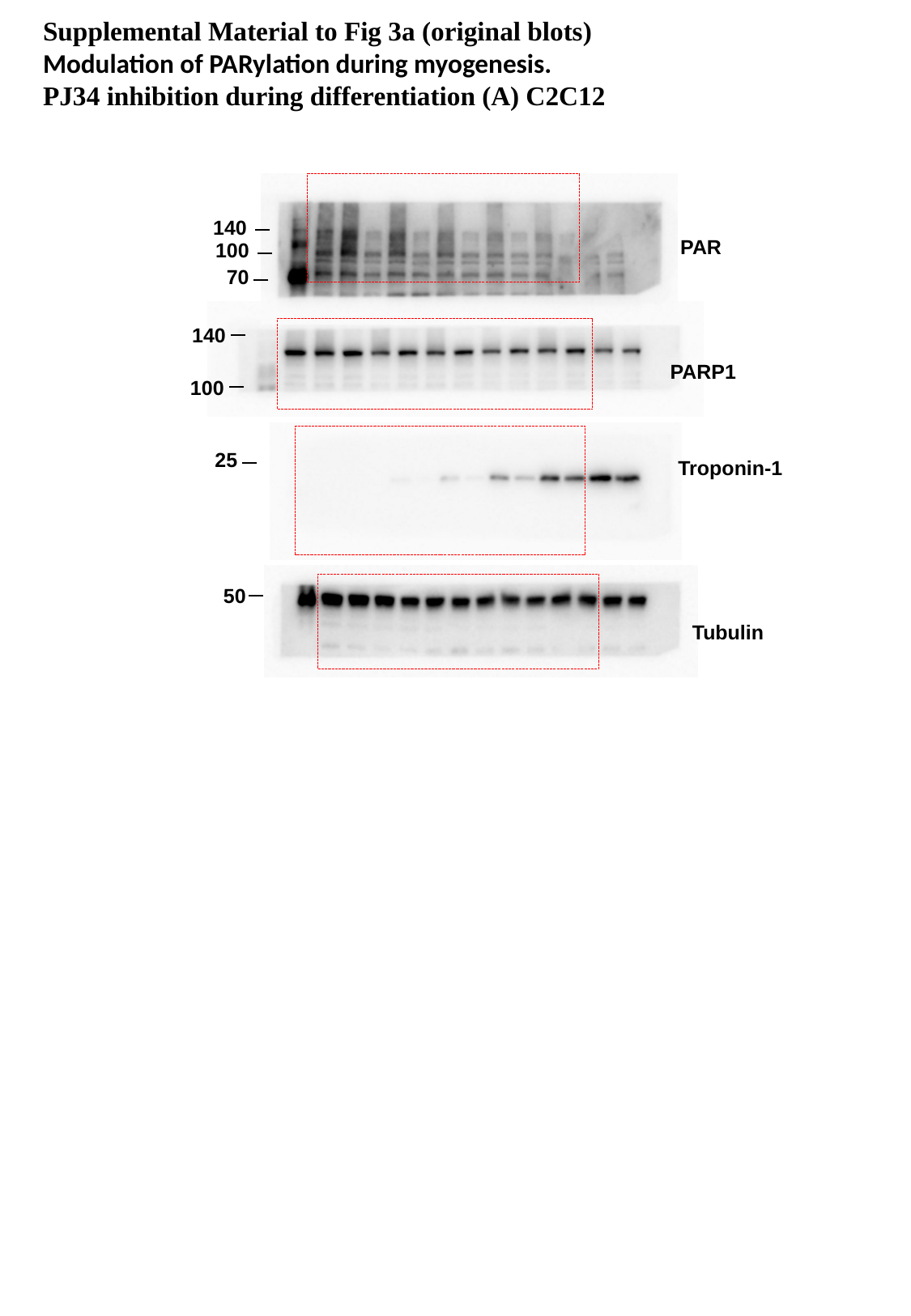

Supplemental Material to Fig 3a (original blots)
Modulation of PARylation during myogenesis.
PJ34 inhibition during differentiation (A) C2C12
140
PAR
100
70
140
PARP1
100
25
Troponin-1
50
Tubulin

## Slide 9
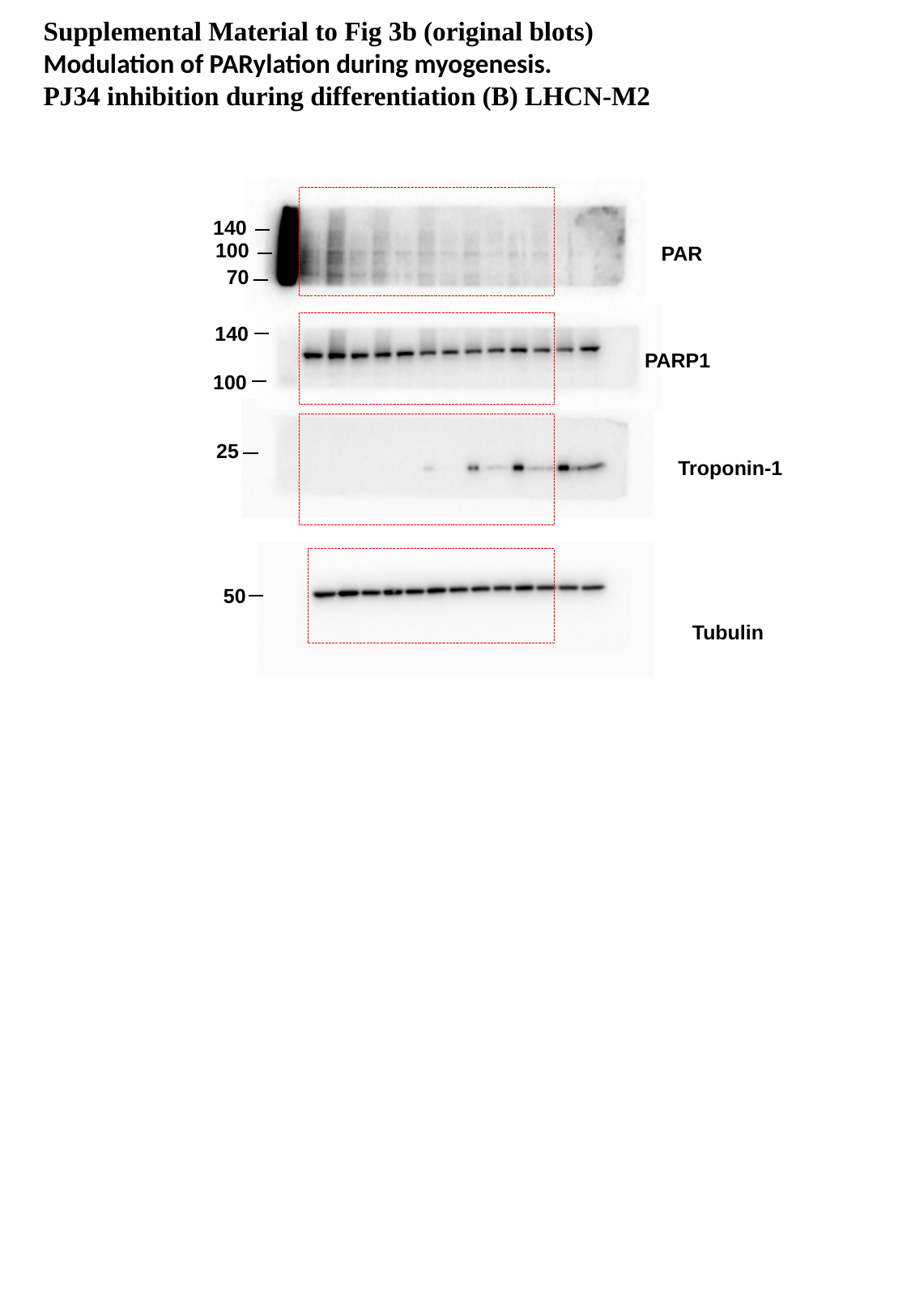

Supplemental Material to Fig 3b (original blots)
Modulation of PARylation during myogenesis.
PJ34 inhibition during differentiation (B) LHCN-M2
140
100
PAR
70
140
PARP1
100
25
Troponin-1
50
Tubulin

## Slide 10
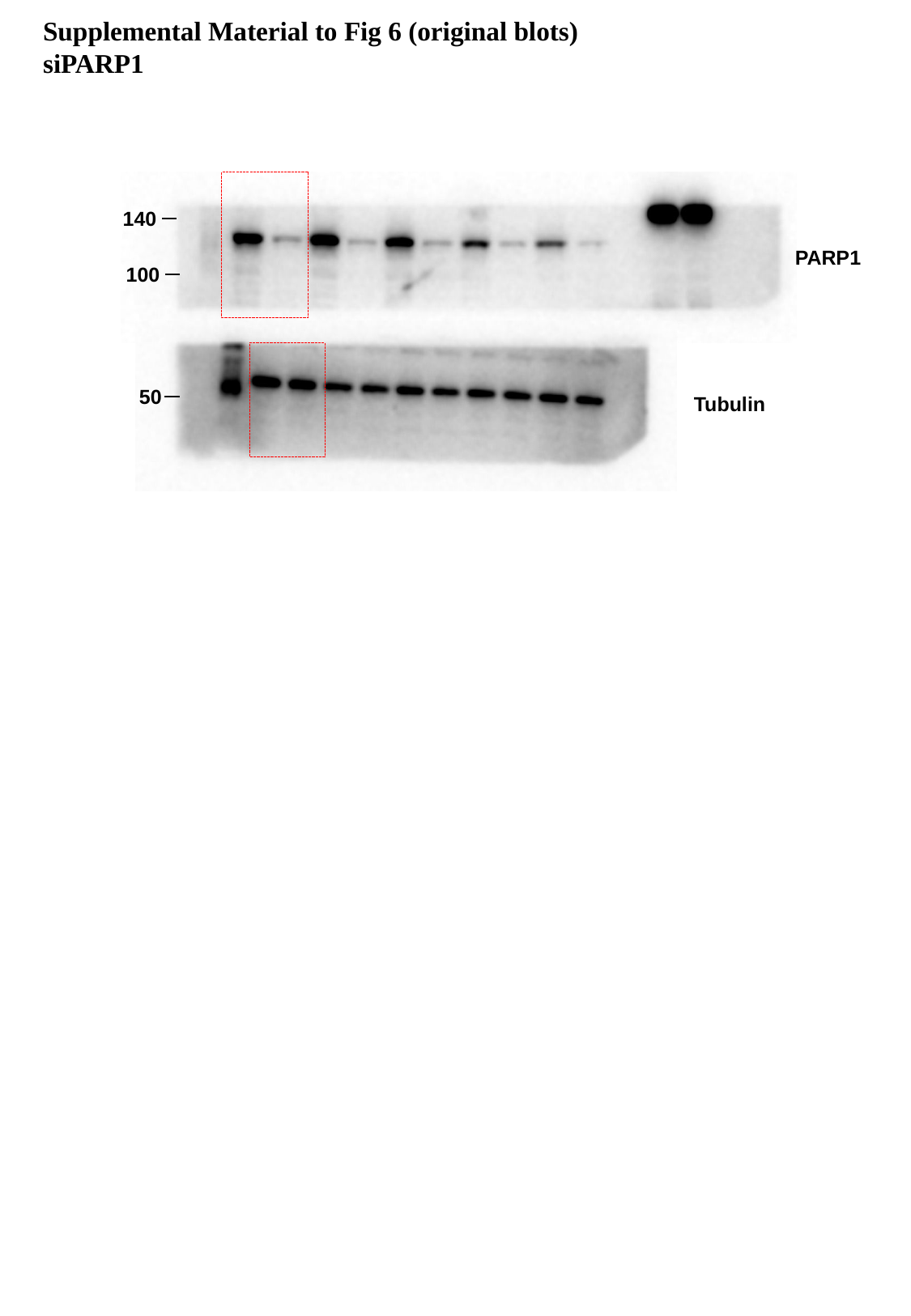

Supplemental Material to Fig 6 (original blots)
siPARP1
140
PARP1
100
50
Tubulin
